# Microfluidic assisted low-temperature and speedy synthesis of TiO_2_/ZnO/GOx with bio/photo active cites for amoxicillin degradation

**DOI:** 10.1038/s41598-022-19406-y

**Published:** 2022-09-15

**Authors:** Somayeh Sohrabi, Mostafa Keshavarz Moraveji, Davood Iranshahi, Afzal Karimi

**Affiliations:** 1 Department of Chemical Engineering, Amirkabir University of Technology (Tehran Polytechnic), Tehran, Iran; 2 Department of Biotechnology, Faculty of Advanced Technologies in Medicine, Iran University of Medical Sciences, Tehran, Iran

**Keywords:** Biochemistry, Chemical biology, Environmental sciences, Materials science

## Abstract

For the first time, a bio-photo-catalyst is synthesized in a microfluidic platform. The microchannel, which is wall-coated by in situ synthesized bio-photo-catalyst is used as an opto-fluidic reactor for amoxicillin degradation. Analyses including SEM, XRD, FTIR, Raman, UV–Vis spectra, and DLS have been used to characterize samples. The structure and morphology of TiO_2_ in microfluidic assisted synthesis are studied at 70–120 °C. The results show that both single-crystalline anatase sample and two-phase samples of anatase and rutile can be attained. According to SEM images, the smallest size and the narrowest particle size distribution (0.86 nm $\pm \hspace{0.17em}0.14$) is achieved by synthesis at 70 °C. Elemental mapping of Ti shows a uniform coating layer on inner walls. Raman signals besides the primary amines in FTIR results show the biological activity of the cross-linked Glucose oxidase (GOx), which is aimed for situ generation of H_2_O_2_. FTIR comparison of bulk and spiral microfluidic synthesized ZnO indicates identical bonds. SEM-coupled with performance experimentation reveal that by regulating the flowrate of spiral micromixer for ZnCl_2_ at 25 µl/min and NaOH at 50 µl/min, the narrowest size distribution and best the bio-photo-catalytic performance of ZnO nanoparticles is observed.

## Introduction

Nowadays, the necessity for large labs and time-consuming steps for nanomaterial synthesis and investigating their applications are omitted by lab-on-chips. Microfluidic devices can improve the synthesis of the nanostructures owing to high control over fluid flow, temperature, mixing, reaction and heat and mass transfer. Multistep processes can be integrated and programmed in a portable chip. A bulk reaction that needs hours to be completed will be done in minutes or less by means of microreactors. In particular, due to the domination of surface forces in microreactors, surface reactions such as photo-catalysis are getting done much faster owing to the improvement of mass transfer resistances, which are dominated in immobilized catalysts^1,2^.

In comparison with bulk reactions, catalytic processes performed in microfluidic reactors possess attractive properties including improved energy efficiency, facilitated recyclability, easy scale-up, and reducibility^3^. Han et al. (2013) have grown ZnO nanowires in a chip and employed the system for methylene blue (MB) degradation. Under the same UV irradiation, the efficiency of the microfluidic system (96%) outranks nearly threefold a bulk reactor (34%)^4^. Zhao et al. (2016) devised a simple, cheap continuous flow ZnO/Zn(OH)F nanofiber arrays-based microfluidic system for the application of photo-catalysis of MB (5 mg L^−1^) as the residence time was 40 s^5^. Meng et al. (2013) presented a novel opto-fluidic microreactor utilizing electro spun nano-fibrous TiO_2_ that fully degrades MB at 53 s and 25 μl/min flowrate^6^.

Cheng et al. (2017) developed an effective photo-catalytic reduction of CO_2_ by using Cu^2+^–TiO_2_ nano-rod thin film photo-catalyst in an opto-fluidic planar reactor under UV light. They conceived that Cu^2+^ ions (1.5 wt.%) were served as electron trapping active sites and could suppress the electron-hole recombination at 80 °C with a flow rate of 2 mL/min, the reaction yield has been maximized^7^. Combining TiO_2_ by carbon-based materials such as nanodiamond (ND) can lower the photocatalyst bandgap and enhance the photocatalytic activity. Though, optimizing their ratio is very important. Y.M. Hunge et al. (2021) have found that TiO_2_/ND ratio of 1:0.75 results in the greatest bisphenol degradations^8^. Bandgap engineering can go beyond the combination of materials, for instance, defect-rich co-catalysts can help the host photocatalyst in creation of more active sites and higher charge separations^9^.

Xu et al. (2015) studied how the H_2_O_2_ oxidant addition affects photo-catalytic, methylene blue (MB) in a microreactor. At 50 min, MB decomposition over Cu@Cu_2_O was 4.7%, while 2 ml H_2_O_2_(30 wt%) without Cu@Cu_2_O, resulted in 75.3% MB, and finally, an efficiency of 96.5% is associated with a case in which both 2 ml H_2_O_2_ (30 wt%) and Cu@Cu_2_O played their role in MB degradation. They attribute the result to the decomposition of H_2_O_2_ to free radicals such as HO˙, HOO˙, and O˙^2−^, which assist the MB degradation^10^.

In view of the cost and hazards of H_2_O_2_ storage, technologies that can lead to its on-site generation and consumption attract the attention of industrial and academic researchers. Glucose oxidase (GOx) bio-catalyzes the oxidation of β-D glucose in the presence of oxygen, resulting in H_2_O_2_ production. The immobilization of GOx is recommended to conquer its carryover^11^. Moreover, the synergy between GOx and TiO_2_ induces a rise in electron transfer rate due to their interaction. For instance, glucose oxidase produces up to 5 times more hydrogen peroxide in the presence of TiO_2_^12^. Municipal, hospital wastewater, and pharmaceutical factories discharge can release micro-pollutants, the emerging contaminants which are hazardous in trace quantities^11^. According to a recent survey, amoxicillin is the high-risk antibiotic in Tehran^13^.

This work is the first report on a facile, low-temperature, and cost-effective synthesis of a bio-photo-catalyst in a microfluidic system. Where ZnO nanoparticles act as dopant of TiO_2_ and GOx enzyme acts as bioactive site that regulates the generation and consumption of H_2_O_2_. A spiral microfluidic mixer is utilized for room-temperature synthesis of ZnO nanoparticles at different flow rates. Meanwhile, TiO_2_ synthesis at low temperatures is done in continuous flow capillaries. For the first time, the illuminated TiO_2_/ZnO/GOx-coated capillary is applied as opto-fluidic reactor for amoxicillin degradation. Amoxicillin has been selected as target pollutant due to its high consumption rate and ecological risk. The effect of amoxicillin initial concentration, TiO_2_ synthesis temperature, and operation time are investigated on the micro-photo-fluidic degradation of amoxicillin.

## Material and methods

### Materials

Titanium (III) chloride (> 15% TiCl_3_ basis, 5–10% free acid as HCl, Sigma-Aldrich) was used in the synthesis of TiO_2_. For ZnO synthesis, zinc chloride (> 98%, Merck) and NaOH (pellets > 97%, Merck) have been used. For rinsing the samples, isopropyl alcohol (> 99.8%, Merck) and double distilled water were used, the latter been prepared in the Research Laboratory at the chemical engineering Department of Amirkabir University of Technology. The Glutardialdehyde solution (25%, Merck), and GOx is gifted from Bonda Group Development. Glucose monohydrates has been employed as the substrate for GOx. The target water contaminant is Amoxicillin trihydrate, which is received from Afa Chimi. Sodium 1, 2-naphthoquinone-4-sulfonate (> 97%, Merck) acts as the chemical derivative chromogenic reagent, K_2_HPO_4_ (> 98%, Merck), and KH_2_PO_4_ (> 99.5%, Merck) for adjusting pH are used in the visible spectroscopic detection of amoxicillin. PDMS (Sylgard 184 Silicon Elastomer Kit, Dow Corning) is used in the microfabrication of the spiral chip.

### Methods

#### Microfluidic synthesis of TiO_2_

**Figure 1:**
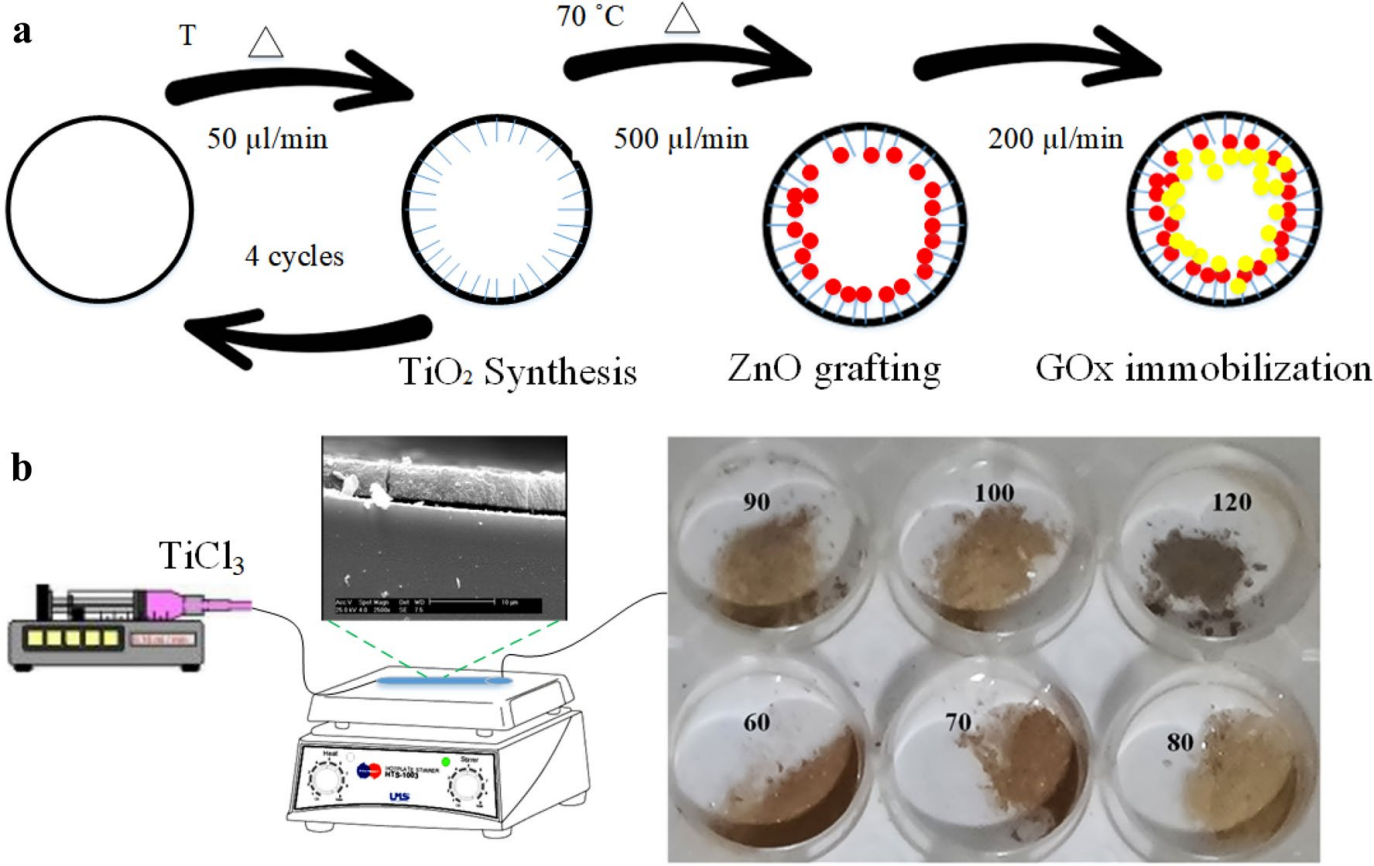
(**a**) GOx/ZnO/TiO_2_ bio-photo-catalyst synthesis steps from the cross-sectional view, (**b**) schematic of the simple facilities used and the products.

According to Fig. 1a, TiO_2_ is synthesized and then ZnO nanoparticles and glucose oxidase enzyme have been immobilized on its surface in two consecutive steps. For this purpose, a solution of TiCl_3_ precursor in water with a concentration of 0.1 v/v at a speed of 1 mm/min, at several temperatures including 60, 70, 80, 90, 100, 120 °C was injected into the capillary tube. These injection cycles have been repeated up to 4 times to devote a total of 100 min for synthesis. To make the coated layer more annealed and durable, the capillary tubes were transferred to an oven and heated at 140 °C for 1 h, and the output solution was dried at the same temperature and some powders were obtained. In Fig. 1b, the real TiO_2_ sample images are also given to highlight the color change owing to the synthesis temperature.

#### Microfabrication of ZnO synthesis chip

During ZnO synthesis, ZnCl_2_ to NaOH streams with concentrations of 30 mM and 50 mM should be mixed in a spiral micromixer, with cross section of 200 µm × 100 µm. The chip is made of PDMS and created on a lamella with plasma bonding, which includes 4 rings with a distance of 2 mm and two Y-shaped inputs and one output. The molding steps are as follows:

1. Combining Silgard 184 with a curing agent at a ratio of 10 to 1 weight and completely stirring.
2. Debubble with a desiccator for 30 min
3. Pour the PDMS mixture into the mold and bake on a hot plate at 90 degrees for 15 min
4. Open and separate the PDMS from the wafer and cut it to the desired size and create the input and output with a 1 mm tissue cutting punch.
5. Cleaning lamellar glass pieces with suitable dimensions with acetone, propanol, and deionized water
6. Drying the glass with clean and dry air
7. Cleaning PDMS with adhesive tape to remove any particles attached to it
8. Placing PDMS and lamellas in the plasma bonding chamber
9. Exposure to plasma with oxygen plasma at a pressure of 8 e-1 m bar, with a power of 40 watts for 1 min
10. Remove the parts and paste the PDMS and glass to each other and heat on a hot plate at 90 degrees for 5 min.

#### Microfluidic synthesis of TiO_2_/ZnO

For the synthesis of ZnO / TiO_2_ nanowires, after the steps related to the synthesis of TiO_2_ nanowires, an emulsion solution containing Zn^2+^ in water with a concentration of 10% by volume for 18 min at a speed of 500 µl/min and temperature of 70 °C. It is injected into capillary tubes coated with TiO_2_. To create this emulsion, a concentrated solution of zinc chloride (65% by weight) containing glycerol (with a weight ratio of glycerol to Zn^2+^ equal to 3.3–1) would be mixed with KOH base solution (50% by weight) at ambient temperature.

#### Microfluidic synthesis TiO_2_/ZnO/GOx

The last section is associated with enzyme fixation on ZnO/TiO_2_. For surfaces activation prior to cross-linking, glutaraldehyde was first injected into the microchannel for 10 min at a speed of 200 µl/min and then GOx enzyme with a concentration of 40 mg/ml in saline phosphate buffer was injected at a rate of 500 µm/min for 4 consecutive cycles with a total of 40 Minutes. GOx/ZnO/TiO_2_ bio-photo-catalyst samples have been placed at 4 °C for 24 h to ensure crosslinking.

### Characterization

The characterization tests include XRD, SEM, DLS, FTIR, UV–Vis and RAMAN spectroscopy. The crystalline phase identification, and crystallite size of TiO_2_ powders, which are collected from the effluents of the capillaries were characterized by X-ray diffraction (XRD) using DIFFRACTOMETER of inel CO. EQUINOX3000 model with Cu Ka radiation: 1.54190 A at 40 kV and 30 mA. The XRD patterns were collected from 5 to 90° in 2ɵ range at a scan rate of 0.032°/s. The crystallite size was calculated from peak broadening using the Sherrer formula. The phases of samples have been detected by means of XPert HighScore Plus software. The morphology of the wall coated TiO_2_ synthesized at temperatures in the range of 70–120 °C, and ZnO nanoparticles, which have been synthesized at different flow rates within a spiral micromixer, were investigated using a scanning electron microscope of Philips Company, XL300 model at the acceleration voltage of 25 kV.

To compare the functional groups of ZnO powder, which is obtained from bulk and microfluidic systems, and to confirm the presence of bio and photo-active cites of the biophotocatalyst, Fourier transform infrared (FTIR) of ZnO, GOx, and TiO_2_/ZnO/GOx have been collected using a Perkin-Elmer Spectrum, Frontier model, Version 10.03.06 (Perkin-Elmer Instruments, Norwalk, CT, USA) in the range of 400–4000 cm^−1^. Raman signals from the biophotocatalyst have been collected using a Raman Microspectrometer in the range of 400–3200 cm^−1^ by a green laser operating at 532 nm with an incident power of 10 mW with a resolution of 16 cm^−1^. DLS (Dynamic Light Scattering) of Cordouan Tech company, VASCO2 model has been utilized to estimate the impact of synthesis temperature on the size of TiO_2_ powders.

### Analytical methods

After the bio-photo-catalysis, DR 3900 spectrophotometer of HACH CO. is used to determine amoxicillin degradation efficiency by a colorimetric method. To 8 mL of amoxicillin, 2.0 mL of 0.2% sodium 1, 2-naphthoquinone-4-sulfonate and 2.0 mL K_2_HPO_4_-KH_2_PO_4_ buffer solution of pH 8.50 are sequentially added. Then, the mixture undergoes room temperature shaking at 150 rpm for 50 min. The blank solution prepared with the same procedure and reagent composition, but no amoxicillin. Finally, visible spectroscopic detection of amoxicillin will be possible at 468 nm, which is much more facile and cost-effective than high-pressure liquid chromatography (HPLC) and more accurate than UV spectroscopy. The method is adopted from^14^ with some modifications. The amoxicillin degradation efficiency has been calculated using the following equation, which is derived from Beer–Lambert law.

## Results and discussions

### Cross-sectional view of TiO_2_ coated microchannels

**Figure 2:**
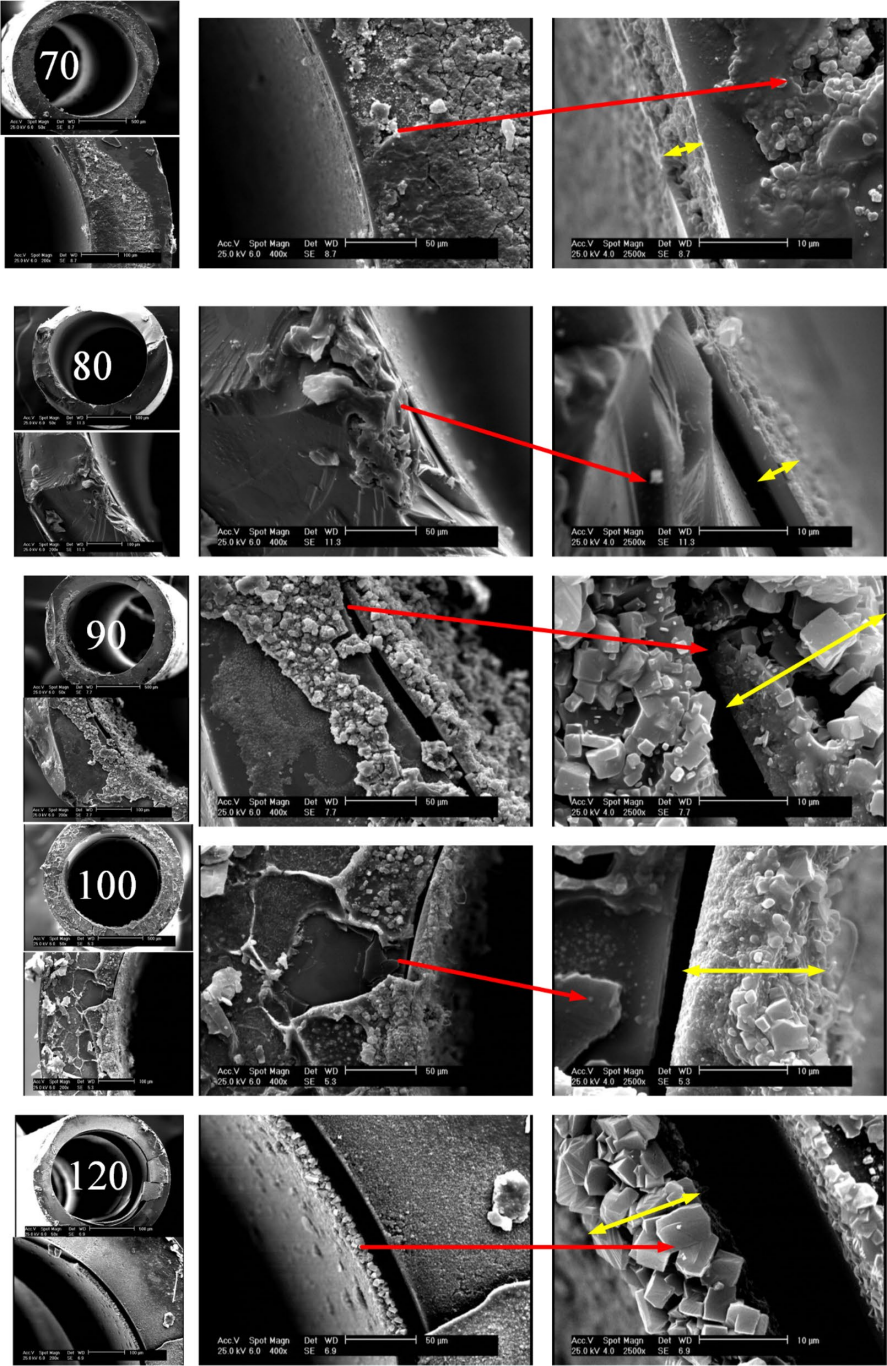
Cross-section SEM images of photo-catalysts grown within the inner wall of the capillaries at 70–120 °C.

To observe how temperature influences the thickness of the synthesized TiO_2_ layer, SEM snapshots of capillaries cross-sections at different scales including 500, 100, 50 and 10 µm and various synthesis temperatures from 70 to 120 °C have been taken. The yellow arrows represent the thickness of the layer. According to Fig. 2, at 70 and 80 °C, the TiO_2_ layer is more uniform and the effect of temperature on the layer thickness is incremental. At 90 °C, particles of different sizes are formed, which causes the thickness of the layer to increase with a greater slope. At 100 °C, due to the initiation of more nucleation, more particles are formed with smaller sizes. At 120 °C, particles with more regular shapes and relatively close sizes are formed, which can be due to the balanced longitudinal and transverse growth of the TiO_2_ structure.

### TiO_2_ growth rate and histogram

**Figure 3:**
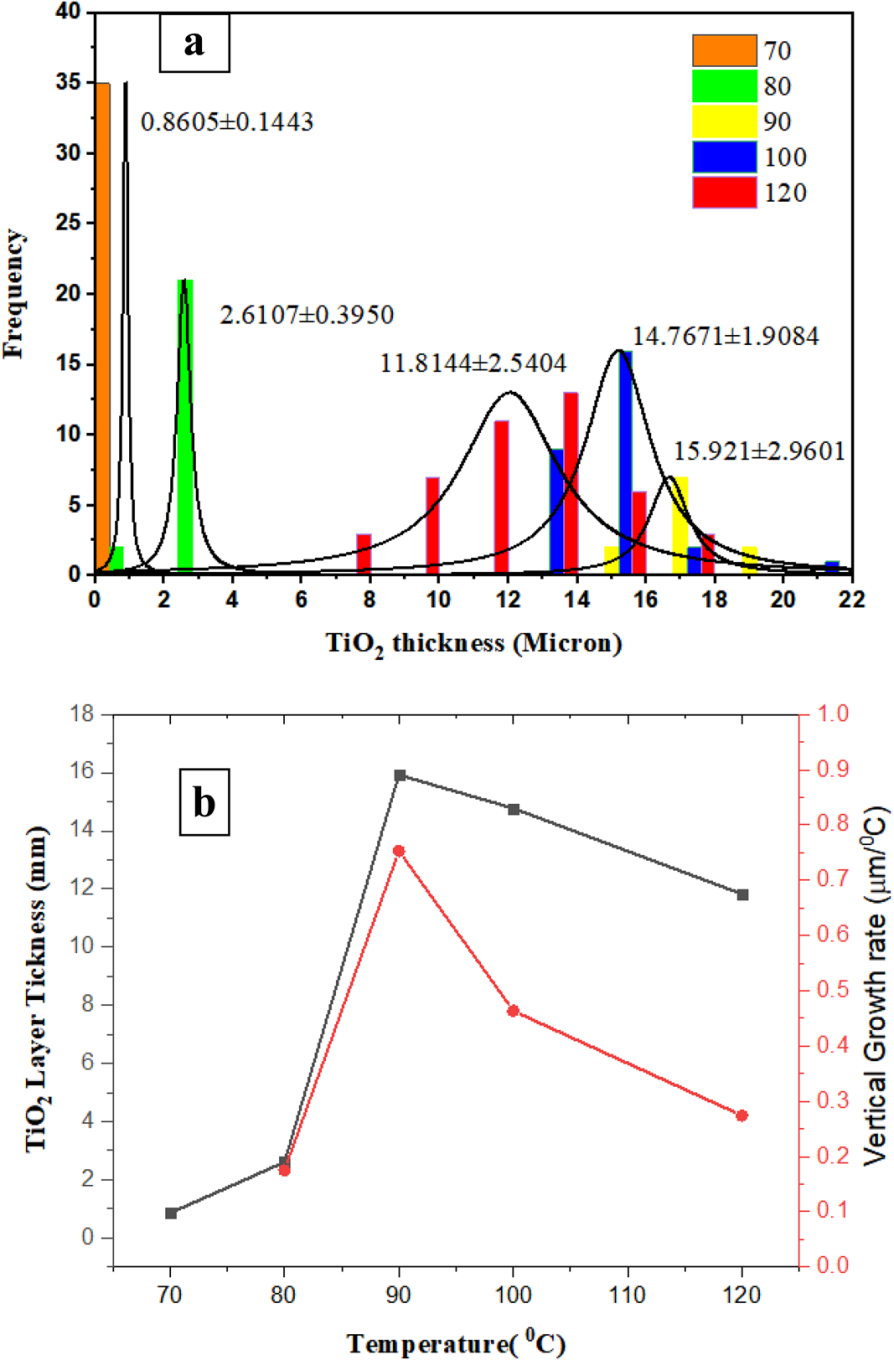
(**a**) The effect of temperature on (**a**) Histogram of TiO_2_ layer thickness, (**b**) vertical growth rate.

According to Fig. 2 and with aid of image j software, the following histogram (Fig. 3a) is attained for TiO_2_ layer thickness, which shows the effect of temperature in the range of 70–120 °C is not inevitably ascending. In other words, from 70 to 90 °C, the layer thickness and its standard deviation increase. From 90 to 120 °C, the layer thickness decreases. At 90 °C, due to secondary nucleation, it is a turning point in microfluidic synthesis. Such behavior was observed in the bulk system at 180 °C. The ideal synthesis temperature can be 70 °C. First, TiO_2_ size is smaller, which is larger according to^15^ corresponding to the specific surface area. Second, size distribution is the narrowest.

Figure 3b shows a graph of TiO_2_ layer thickness increase and longitudinal growth. Growth rate ((L_T_-L_70_)/∆T) is considered based on the rate of the average thickness increase at each temperature relative to the average layer thickness at 70 °C divided by its temperature difference with 70 °C.

Due to the increase in temperature from 70 to 80 °C, the layer thickness increases from 860 to 2.61 mm. When the synthesis temperature increases from 70 to 90 °C, the layer thickness reaches 15.92 µm, and the growth rate increases more than 4 times. Once the synthesis temperature increases from 70 to 100 °C, the layer thickness reaches 14.77 µm, and the growth rate increases more than 2.5 times. With the synthesis temperature increasing from 70 to 120 °C, the layer thickness reaches 11.82 µm, and the growth rate increases more than 1.25 times.

### Surface study of the inner wall microchannel

**Figure 4:**
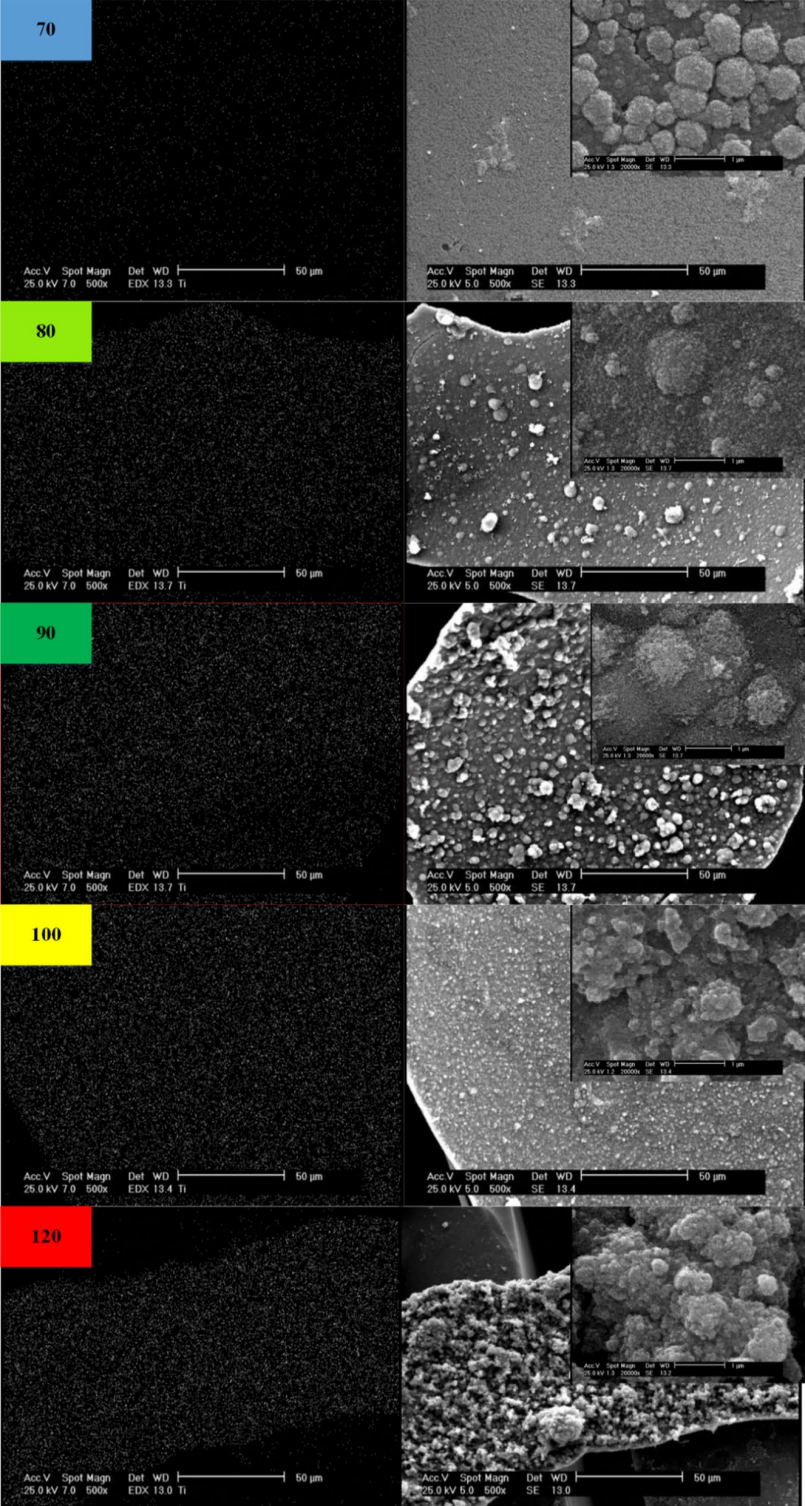
Surface morphology of TiO_2_ and Ti Elemental mapping at several temperatures.

According to the SEM images shown in Fig. 4, the synthesis at 70 °C resulted in the production of 50 nm particles that are homogeneously dispersed on the surface. A few nanoclusters with a maximum diameter of 700 nm are also witnessed, but they are much smaller in size and frequency compared to the ones detected at higher synthesis temperatures. As the temperature increases, a number of secondary nuclei can be seen, which peaks at 90 °C. At 80 °C, the presence of several particles about 5 microns in length is seen, indicating an increase in the particle size distribution range. At 90 °C, the number of larger particles is greater. At 100 °C more particles are formed with smaller sizes. At 120 °C high surface porosity and particle growth can be seen. EDX results confirm the uniformity of the coating and the presence of titanium dioxide.

Hurtado et al. 2016 proved that the photonic efficiency is two orders of magnitude higher in the coated capillary reactor than in the slurry stirred reactor (STR), and it is twice slurry capillary^16^. It should be noted that an aqueous solution is forced through a micro-packed bed capillary, it will tend to circumnavigate the beads instead of passing through them, which affects the yield of the process^17^.

### How synthesis cycles affects TiO_2_ size

**Figure 5:**
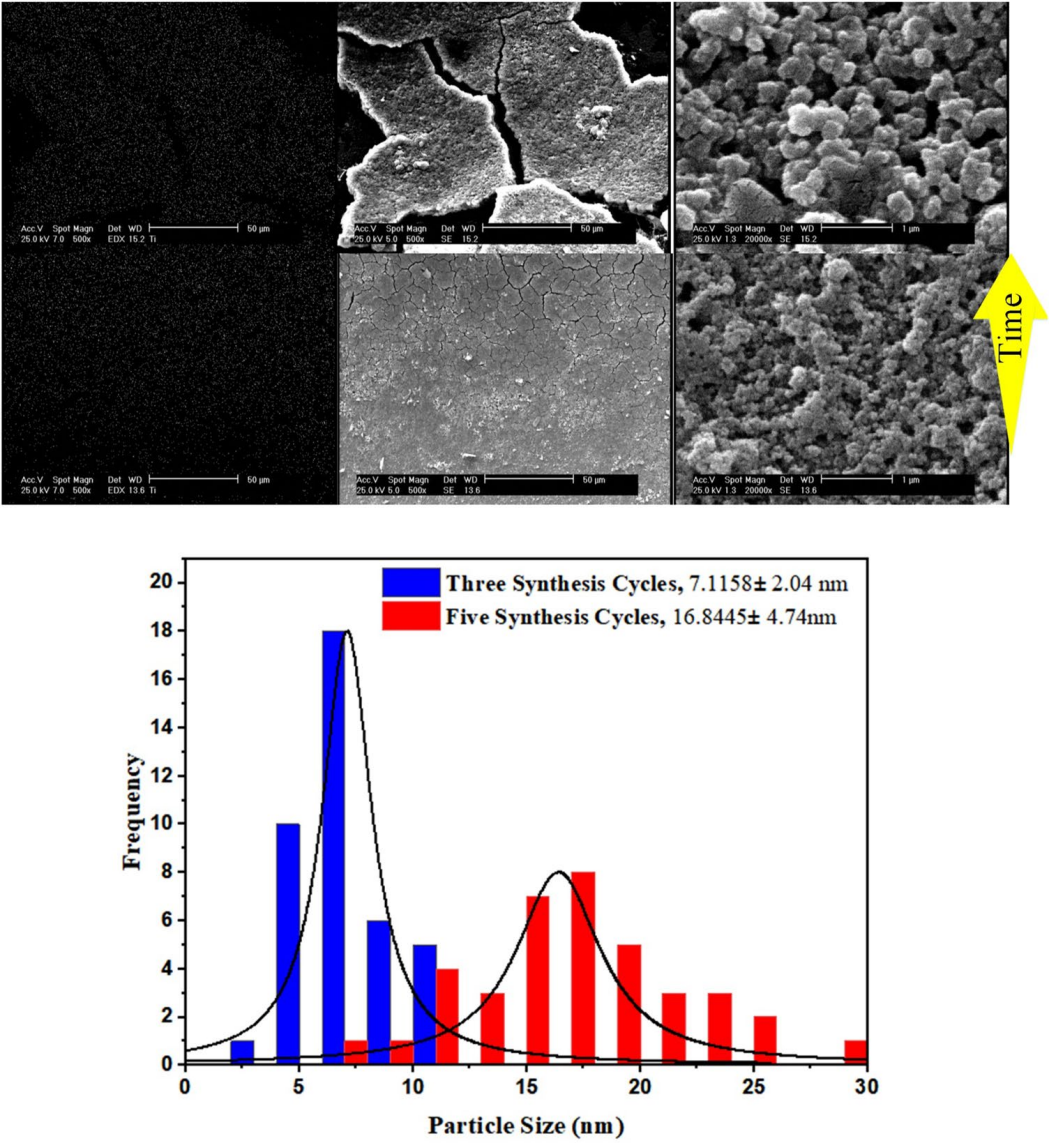
The impact of synthesis time on the microchannel surface coverage and size of TiO_2_.

To investigate the effect of synthesis time on morphology, following the synthesis procedures, the only variable was the synthesis time. Therefore, the precursor solutions are injected into the capillaries for three and five consecutive cycles at 80 °C. A total of 75 and 125 min were devoted to synthesis, and then the capillaries are transferred to a conventional oven and heated at 140 °C for 1 h to make the coated TiO_2_ layer durable. As shown in Fig. 5, the particle size increases with increasing synthesis time, as well as the cracks on the surface.

The histograms indicates that synthesis time of 75 min leads to TiO_2_ mean size of 7.11 nm with 2.04 nm standard deviation. Once synthesis time increases to its 1.67fold, both the particle size and size distribution proliferates more than two times.

### Crystallographic study of TiO_2_ powder

**Figure 6:**
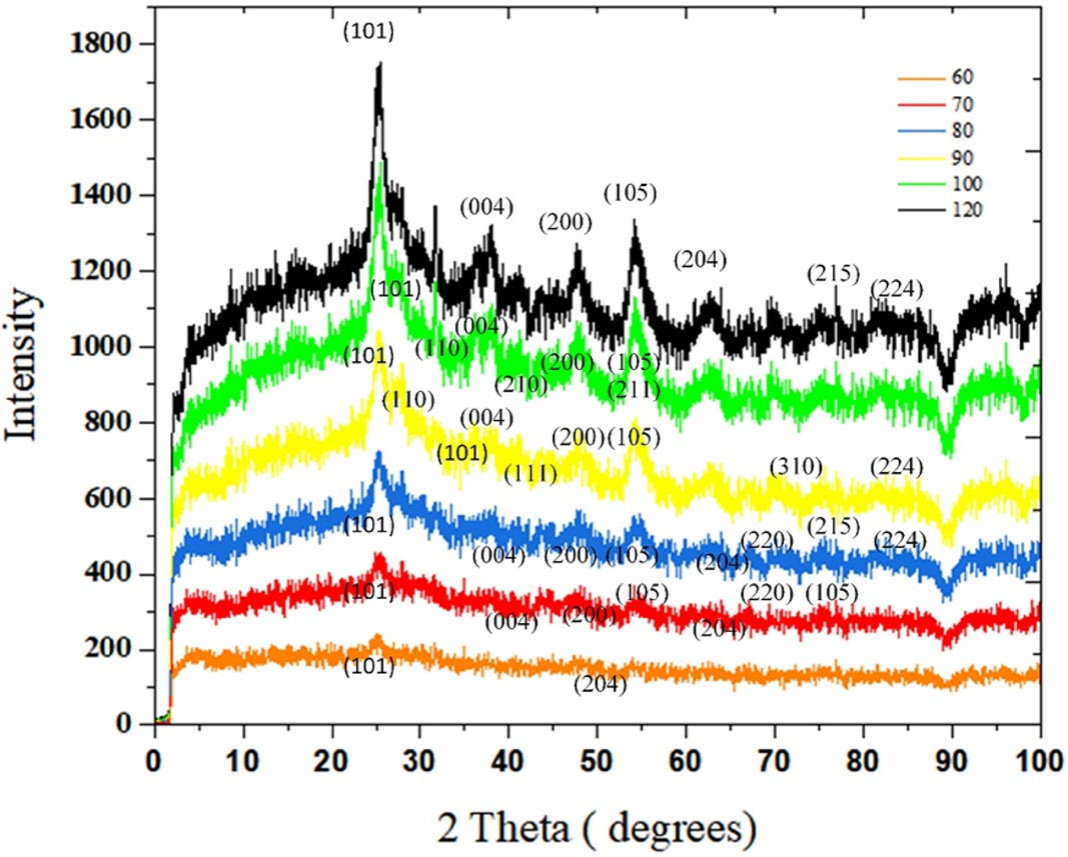
The impact of synthesis temperature on crystalline phases of TiO_2_ samples in the microfluidic system.

The XRD spectrum of samples synthesized at different temperatures in the range of 60–120 °C is given in Fig. 6. It is noted that these samples are collected from the effluents of the capillaries. With synthesis temperature of 60 °C, two anatase peaks of (101) and (200) planes appear. As the temperature rises to 80 °C, the sum of peaks proliferates. (7 peaks for 70 °C and 8 peaks for 80 °C haven been detected). For 70 °C, Anatase peaks at 25.5°, 38.3°, 48.9°, 54.5°, 63.6°, 71.3°, and 76.3° are associated with (101), (004), (200), (105), (204), (220), (215) crystalline planes^18^. Such peaks are also observed for the sample synthesized at 80 °C, in addition a new peak at 82.1° (224) (Anatase XRD JCPDS Card no. 78-2486).

Both phases of anatase and rutile are detected in the spectra of the synthesized samples at 90–100 °C. At 90, Five anatase peaks which are related to (101), (004), (200), (105), and (224) planes are seen, plus four peaks newly formed rutile peaks of 27.4°, 36.3°, 41.1°, 69.8°, which ascribes to (110), (101), (111), (310) ^19,20^. Once TiO_2_ is synthesized at 100 °C, as well as (101), (004), (200), (105) crystalline anatase planes, three rutile peaks have been witnessed at 31.7° (110), 45.5° (210), 54.3° (211)^20–22^. In the last spectra, which is related to the sample synthesized at 120 °C, the intensity of peaks has reached the highest quantity. Anatase peaks associated with (101), (004), (200), (105), (204), (215), (224) crystalline anatase planes^18,19^.

X_A_ (%) is the weight percentage of anatase the samples which consists of both anatase and rutile phases. It is calculated from the following equation:

1 $${\text{XA }} = \frac{100}{{\left( {1 + 0.26 * \left( {IR / IA} \right)} \right) }}$$

where "I" represents the maximum peak intensity, A represents anatase and R denotes rutile. The weight percentage of rutile is obtained by subtracting the weight percentage of anatase from 100. According to the XRD results, from 60 to 80 °C, the anatase phase is formed. Increasing the synthesis temperature from 80 to 100 °C has led to partial conversion of anatase to rutile phase. To calculate the crystallite size (D), the Scherer equation (Eq. 2) is used.

2 $$D=\frac{K\lambda }{\beta COS\theta }$$

where K is the Scherer constant (correction factor related to the sample shape and is equal to 0.9, λ beam wavelength, β peak width at half the maximum height, θ is the diffraction angle.

Average crystallite size (${D}_{ave}$) can be resulted from Eq. (3) ^23^:

3 $${D}_{ave}={D}_{A}\left(\frac{{I}_{A}}{{I}_{A}+{I}_{R}}\right)+{D}_{R}\left(\frac{{I}_{R}}{{I}_{A}+{I}_{R}}\right)$$

Table 1 shows that as synthesis temperature rise, crystallite size increments ^24,25^, but once new crystalline phase is being formed, a decrease in average crystallite size is observed. From 60 to 70 °C, anatase single crystallites grow. The onset of rutile phase formation is 80 °C, the crystallite size gets a bit smaller. The crystal size reduced caused by the burst of nucleation was more palpable than that of the anatase crystal growth accelerated caused by the increase of temperature^26^. At 90 °C, a considerable quantity of anatase and rutile nucleons with a very small average crystallite size of 8.84 nm have been formed. Increasing the temperature up to 100 °C, the average crystallite size has been doubled. Moreover, in multiphase samples, by increasing the rutile/anatase ratio (0.97–1.17) and increasing the synthesis temperature (90–100 °C), the average crystallite size increases from 8.84 to 18.9 nm. A similar trend is reported in^27^, where decreasing anatase/rutile ratio from 5.4 to 0.25, and synthesis temperature from 55 to 90 °C, the average crystallites grow from 33.16 to 88.44 nm^27^. The synthesis temperature of 120 °C leads to pure anatase nucleation and growth, and crystallite size reaches 88.68 nm.

**Table 1:** Phase and crystallite size of TiO_2_.

Synthesis Temperature (°C)	Phases			Crystallite Size (nm)		
	X_A_ (%)	X_R_ (%)	Ratio (R/A)	D_A_	D_R_	D_ave_
60	100	0	0	88.40	0	88.40
70	100	0	0	97.20	0	97.20
80	100	0	0	89.22	0	89.22
90	50.76	49.24	0.97	6.15	30.98	8.84
100	46.05	53.95	1.17	7.68	12.32	18.9
120	100	0	0	88.68	0	88.68

### DLS study of TiO_2_ powder

To have an estimate of the size of TiO_2_ powder, a series of dynamic light scattering tests have been performed. For sample preparation, a solvent is needed, herein, isopropyl alcohol is used as the liquid medium with a refractive index of 1.36 and viscosity of 1.057 cP. The Pade Laplace algorithm is considered. The findings are summarized in Table 2, as the synthesis temperature rises, the diameters of nanoparticles decrease, and the diffusion coefficient increases. The results are in agreement with Figs. 3 and 4, which notify that near-wall TiO_2_ nanoparticles become bigger with temperature increase, and therefore, the centered nanoparticles have a descending relationship with temperature. Likewise, with the immobilized TiO_2_, a sudden increase of size at 90 °C is observed.

**Table 2:** DLS results about synthesis temperature-size relation of TiO_2_.

Synthesis Temperature (°C)	Mean ( nm)	Diffusion Coefficient (m^2^/s)
60	631.67	65.38855E-014
70	614.18	67.25027E-014
80	586.3	70.44867E-014
90	610.04	67.70725E-014
100	585.24	70.57533E-014
	17.97	22.98924E-012
120	430.44	95.95805E-014

### Identification of ZnO nanoparticles

In order to compare the results of the microfluidic system with bulk, the molar ratio of ZnCl_2_ to NaOH is kept constant at 0.6 with concentrations in the microfluidic system for the first and second streams are 30 and 50 mM, respectively (50 times dilution of the bulk systems). A microfluidic platform for synthesis comes along with more control over reaction, in the meantime, the reduction of chemicals consumption strongly prevents the occurrence of chemical accidents. Especially for highly acidic or basic circumstances. Synthesis time in room temperature spiral microfluidic system has been performed in 15 min V.S 2 h at 100 °C for the bulk approach.

**Figure 7:**
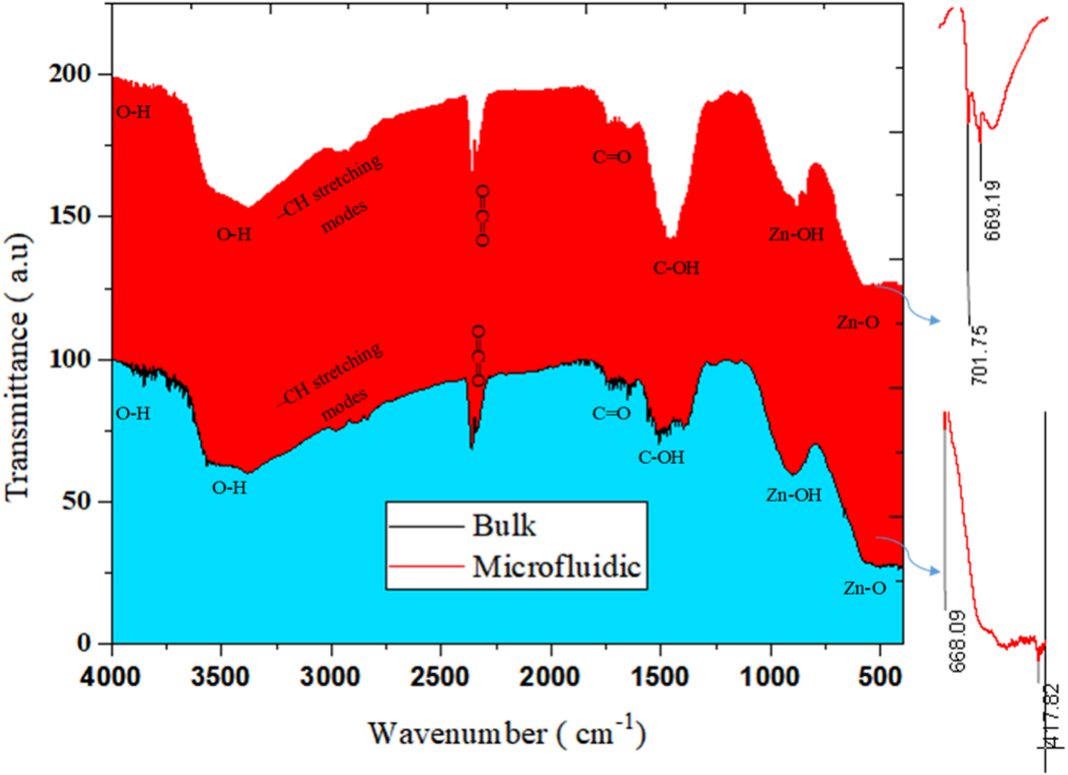
Bulk and microfluidic synthesis of ZnO.

According to Fig. 7, the results of FTIR analysis associated with ZnO synthesis in the bulk and microfluidic system state the existence of identical peaks in the two samples. ZnO stretching vibrations are observed for both methods in the range of 400–668 cm^−1^^28^. In the range of 870–1000 cm^−1^, there are Zn–OH peaks that are higher in number and intensity for nanostructures synthesized by the microfluidic method. In the range of 1200–1500 cm^−1^, the C–OH bond, which can be formed as a result of the OH group due to washing with alcohol, indicates the bending vibration within –OH group in plain^29,30^. These peaks are seen more in the sample of the bulk method. Carboxylic group (C=O) was observed around 1700 cm^−1^ ^31,32^. A peak can be detected at 2300 cm^−1^, which indicates the atmospheric absorption of CO_2_^33^. In the range of 2900–2800 cm^−1^, it shows the peaks related to asymmetric and symmetric stretches of –CH and –CH_2_ groups. Peaks attributed to OH group stretching vibration have been identified at wave numbers higher than 3600 cm^−1^. The presence of O–H stretches and hydrogen bonding by alcohol or water molecules can be found in peaks at 3400 or 3300 cm^−1^ ^34,35^.

### The effect of precursors flowrate on ZnO size

The aim of this section is to investigate the morphology of ZnO nanoparticles synthesized in the spiral system at room temperature. Since the mixing in this type of microreactor is ultrafast, rapid nucleation is expected. A straightforward way is to tune the flow rate which influences the interface concentrations. Therein, the effect of the volumetric flow rate of ZnCl_2_ and NaOH streams are considered in three cases (1) both: 25 µl/min (2) both: 50 µl/min (3) zinc chloride: 25 µl/min and sodium hydroxide flow: 50 µl/min.

**Figure 8:**
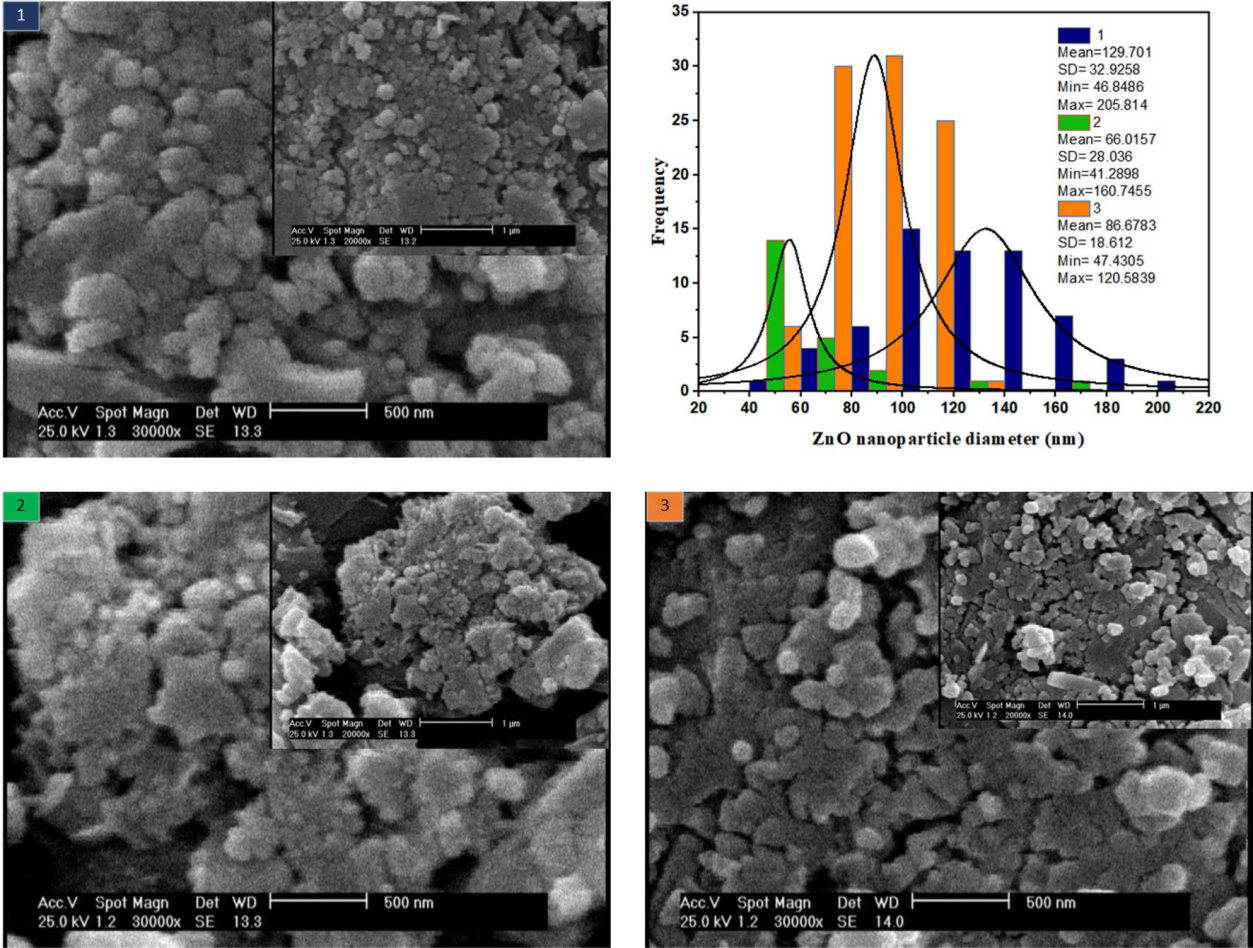
Effect of reagents volumetric flowrate on the morphology of ZnO nanoparticles room temperature synthesis in spiral microreactor.

SEM illustrations in Fig. 8 are provided in two magnifications of 500 and 1 µm. In the 1st case, where the volumetric flow of the two streams is low and equal, the residence time is high, leading to larger particles and a broader ZnO size distribution. In the 2nd case, when the flow rate doubles compared to the first case, the nanoparticle size is almost halved and the ZnO size distribution is somewhat reduced. In the 3rd case, where the ZnCl_2_ flow rate is equal to 25 µl/min and the NaOH flow rate is equal to 50 µl/min, the particle size is intermediate. In addition, the narrowest size distribution is related to this case.

### Biological activity of immobilized enzyme

**Figure 9:**
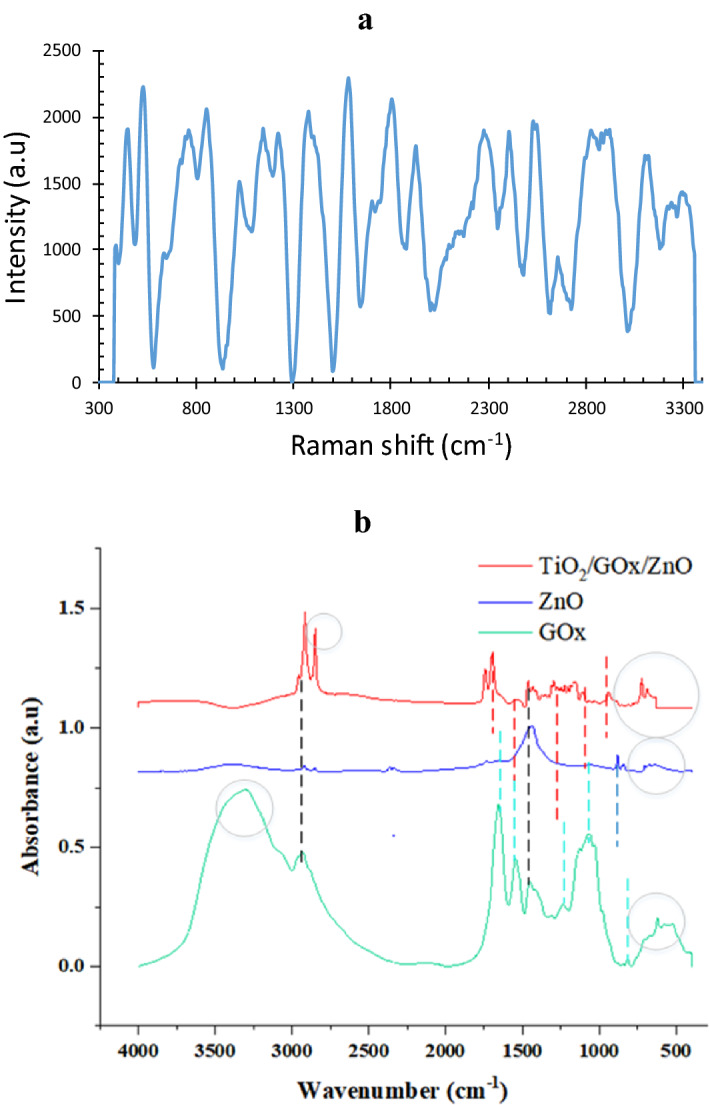
(**a**) Raman spectra of GOx/ZnO immobilized on TiO_2,_ (**b**) FTIR analysis of the bio-photo-catalyst components.

As an instance, Raman analysis of bio-photo-catalyst with a GOx/ZnO ratio equal to 0.25 is shown in Fig. 9a some assignments of Raman vibrations modes of GOx are shown in the spectrum, which are in agreement with^36^. The results evidently validate that the remarkable SERS activity of the fabricated bio-photo-catalyst is induced by the synergistic effects of the plasmonic Zn^2+^^37^, semiconductor TiO_2_, and glucose oxidase molecules, not only induced by plasmonic metal Zn nanoparticles. ZnO lattice vibration is associated with a sharp peak at 454 cm^−1^^38^. At 532 cm^−1^ corresponds to the oxygen vaccines in the TiO_2_/GOx/ZnO. The peak at 856 cm^−1^ is corresponding to TiO_2_^39^. Moreover, the bioconjugation of ZnO/GOx ^40^ and the bandgap reduction of TiO_2_ as a result of doping with ZnO may affect the Raman shift. Pure glutaraldehyde has a strong band at 1125 cm^−1^ ^41^ that originates from C−OH stretching. However, crossed linked GOx showed a band at 1146 cm^−1^. This shifting could be explained by the conjugation of TiO_2_ and ZnO restricting the vibrations of oxygen.

The results of FTIR analysis for bio-photo-catalyst components are shown in Fig. 9b. In the ZnO sample, ZnO tensile vibrations were determined with peaks of 701 and 669 cm^-1^^28^. In the TiO_2_/GOx/ZnO sample, a peak at 668 cm^−1^ could correspond to Ti–O–Ti and ZnO^42^. Two peaks in the range of 900–800 cm^−1^ refer to Zn–OH^29,30^. Peaks 939, 1101, 1159, 1206, 1226, 1297 cm^−1^ are related to Zn-OH and Ti–OH^42^. The presence of this bond indicates hydrolysis of the precursor ^43^. For glucose oxidase, a peak at 1062 cm^−1^ was observed for the stretching vibrations of the C–O bond. In the meantime, this peak with a decreased intensity is detectable in the bio-photo-catalyst Peaks in the enzyme in the range of 1300–1400 cm^−1^ can also belong to the phenolic group of glucose oxidase^44^. In the bio-photo-catalyst spectrum, the peak in this region is also observed.

Two types of peaks were observed in all three samples. The presence of peaks in the range of 1400–1500 cm^−1^ may be related to the (CH)n bonds in the fatty acid or enzyme. Moreover, the C−H stretching modes of α-carbon and the aliphatic carbon chain of glutaraldehyde could be assigned near 2800 cm^−1^ ^41,45,46^.

In the spectrum related to glucose oxidase enzyme, the presence of peaks related to amides of the first, second, and third types of peptide structure is also observed. At 1242 cm^−1^ and 1244, which are related to C–N and C–H tension and N–H torsion^47^. Receptor bonds of the first type were observed at 1600 cm^-1^ to 1700, which are related to the tensile vibration of C=O or CO peptide bonds in the protein structure, second amide bonds at 1500–1600 cm^−1^, NH tension, and C–N tension of the peptide groups. Primary amines are commonly used to monitor structural changes in proteins, and this shows the biological activity of GOx^48,49^. These peaks coexist in the bio-photo-catalyst, however, compared to pure glucose oxidase, their intensity is diminished due to the bioconjugation. There is also a peak at 1743 cm^−1^ and 1699 which belongs to the carbonyl group^50^. In addition, GOx shows amide-related bonds at 3400–3440 cm^−1^ and amide B at about 2900 cm^−1^, which originate from the Fermi resonance between the first peak of amide II and the N–H tensile vibration^51^. The peaks observed in the two samples ZnO and GOx in the range of 3300 cm^−1^ indicate the presence of surface water and O–H bond^42,46^. This peak confirms the presence of hydrophilic spots^31,32^.

### Optical study

**Figure 10:**
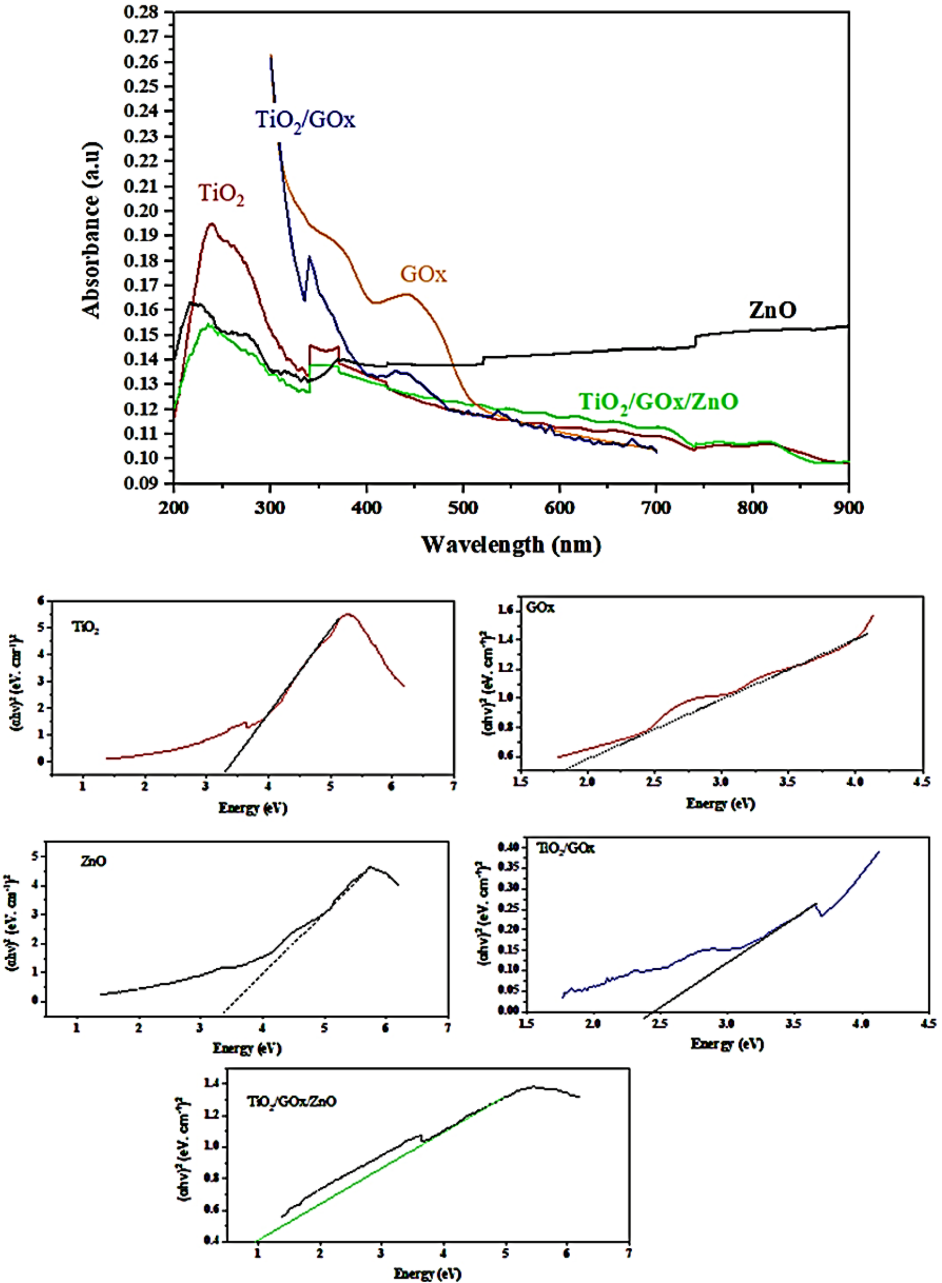
UV–Vis DRS spectra and indirect transition band gaps.

UV–Vis diffuse reflectance absorption spectra (DRS) of TiO_2_, GOx, ZnO, TiO_2_/GOx, TiO_2_/GOx/ZnO in the range of 300–700 nm are shown in Fig. 10. Anatase TiO_2_, which is synthesized at 70 °C is utilized in this test. For TiO_2_/GOx, a shift of the absorption edge to the visible light region is witnessed. After 420.6 nm, the adsorption intensity ascribed to TiO_2_/GOx/ZnO is higher than bare TiO_2_.

The Tauc method is based on the assumption that the energy-dependent absorption coefficient α can be expressed by (αhν)^1/2^ or (αhν)^2^ = B (hν-E_g_) where h is the Planck constant, ν is the photon’s frequency, Eg is the band gap energy, and B is a constant^52^.The corresponding band-gap energy, calculated by using the Kubelka–Munk (KM) method and the plot of (αhν)^2^ versus the photon energy (hν) for pure TiO_2_ and ZnO are 3.20 and 3.33 eV, respectively. ZnO/TiO_2_ heterojunction composite fibers has a bandgap of 2.9 eV^53^. Binary composite of TiO_2_/GOx has a bandgap of 2.43 eV, while for the TiO_2_/GOx/ZnO, bandgap is as low as 1.00 eV. Triple-heterojunction can improve the migration of photo-excited charge carries among different components to enhance photo-activity and charge separation^54^.

### Bio-photo-catalytic degradation of amoxicillin

Amoxicillin synthetic wastewater with concentrations of 10–50 ppm is injected into the bio-photo-catalyst coated microchannel at a speed of 0.5 mm/min. The multiphase reaction has been performed for 1 h under UV light irradiation (0.25 W/cm^3^).

**Figure 11:**
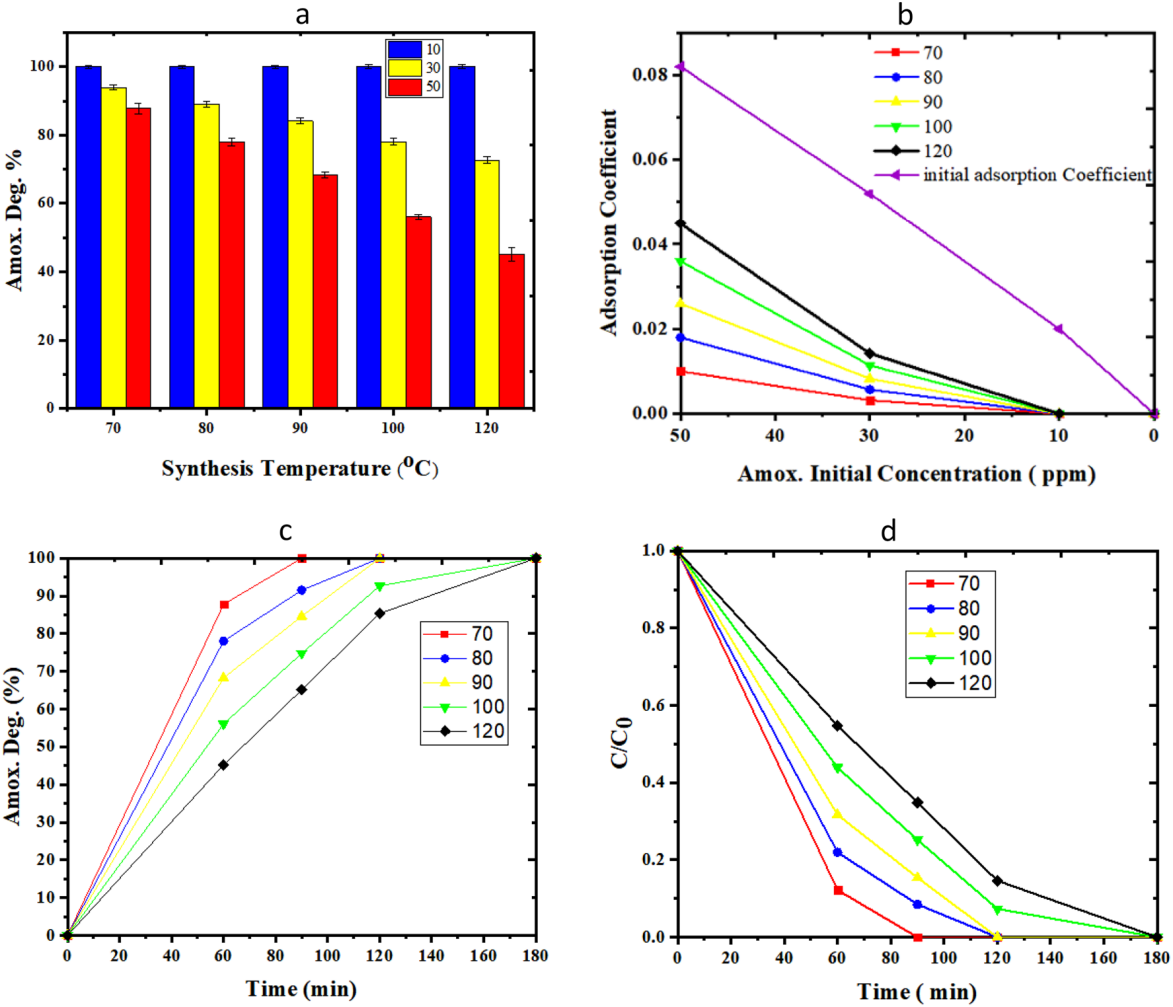
Evaluation of TiO_2_/ZnO/GOx bio-photo-catalyst assay in Amoxicillin degradation, (**a**, **b**) the effect of synthesis temperature and initial amoxicillin (10–50 ppm) degradation over 1 h, (**c**) the effect of operational time on amoxicillin degradation (0–180 min, initial concentration of 50 ppm), (**d**) C_Amox._/C_Amox., 0_ verses time.

Figure 11a investigates the effect of synthesis temperature (70–120 °C) on the bio-photo-catalyst activity. In the whole range of synthesis temperature, can fully degrade amoxicillin up to 10 ppm. Figure 11a makes clear that at the same operational time, the lower concentration of amoxicillin and the lower synthesis time of TiO_2_, the higher the decomposition yield. The more pronounced adverse impact of increments in synthesis temperature rise can be observed in cases with great initial concentrations of amoxicillin. The effect of temperature can be interpreted by the SEM results of photo-catalysts synthesized in the microfluidic system. The photo-catalysts which are synthesized at 70 °C have the smallest diameter and length and size distribution. Figure 11b gives more details on the decrease of amoxicillin adsorption coefficient at different initial amoxicillin concentrations. Figure 11c, d study the effect of time on amoxicillin degradation over the bio-photo-catalysts with synthesis temperatures of TiO_2_ in the range of (70–120 °C). According to the pseudo first-order reaction, the plot of ln(C_0_/C) against time should be linear. Kinetics of reaction reveal that apparent rate constants for samples (70–120 °C) are as 0.035, 0.027, 0.0206, 0.0206, 0.0152 min^−1^, respectively. In the meantime, the behavior of the sample with 70 °C synthesis temperature defines with the highest yield and most rapidly degradation of amoxicillin.

**Figure 12:**
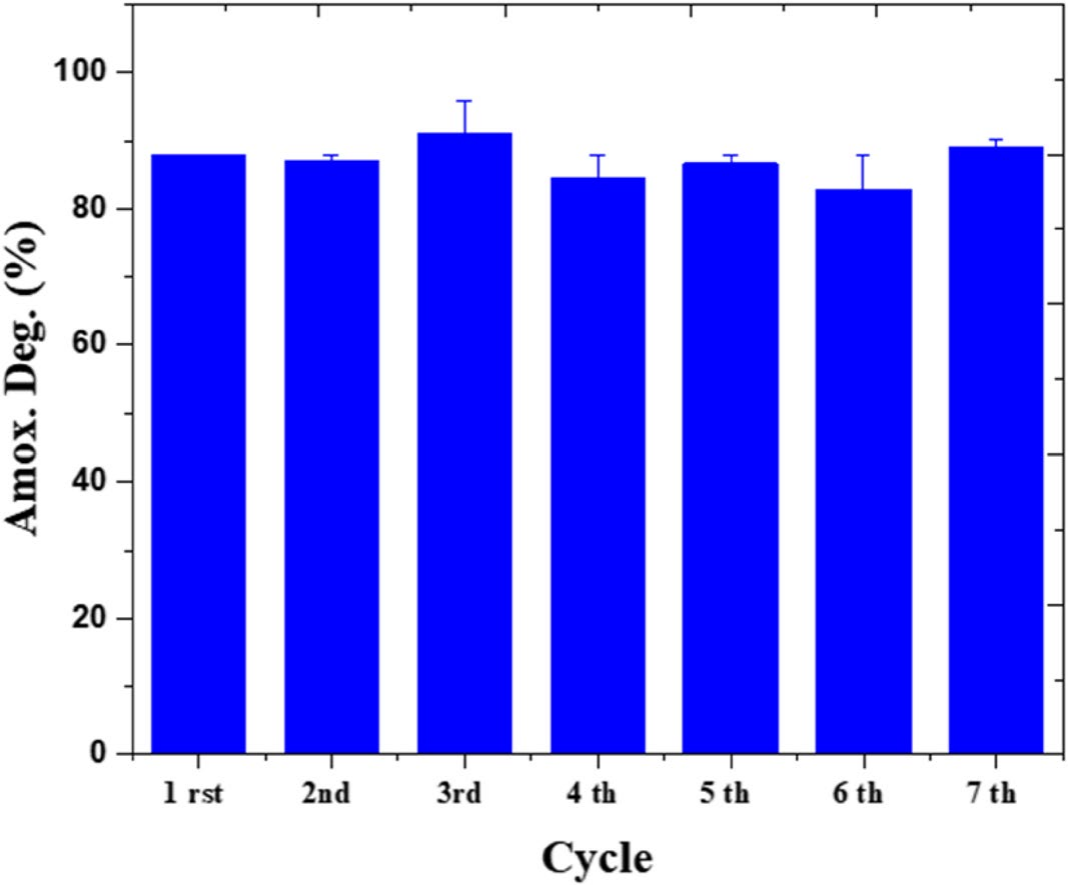
Repeated use of the biophotocatalyst for amoxicillin degradation.

For stability test, amoxicillin solution of 50 ppm illuminated by 0.25 W/cm^3^ is fed up into the microreactor at 0.5 mm/min. In these series of experiments, the operational stability of the system in seven consecutive cycles is studied. The residence time was 1 h and no rising has been done between cycle intervals. In this work, only 8.1% alteration is seen in the amoxicillin degradation efficiency (Fig. 12). Similar results have been observed in where tetracycline is to be degraded in a microfluidic systems over ZnO/ZnS by 5% change in the efficiency after 5 cycles^55^. Microfluidic reactors provide uniform conditions of light propagation and flow distribution, which result in homogenous photoactivation of sites, regular interaction between active sites and pollutants, and eventually these systems allow higher stability compared to bulk reactors.

Table 3 summarizes the published articles on the photo-catalytic amoxicillin degradation. In a recent work, the use of commercial titanium dioxide immobilized on the membrane to decompose amoxicillin (50 ppm) is reported in which, it is removed up to 80% after 500 min^56^. In another work, a hybrid nanostructure of TiO_2_/WO_3_ with a calcination temperature of 700 °C has been utilized at a dose of 0.1 g/l to decompose 25 L of amoxicillin (100 ppm) and the intensity of UV solar radiation was set to a constant value, on a pilot scale. They achieved an efficiency of 64%^57^. TiO_2_ as a slurry of titanium dioxide (anatase) at a reaction time and light intensity less than the previously stated articles, showed a 70% efficiency in the decomposition of amoxicillin, but its separation from the solution for reuse of photo-catalysts is more difficult^58^. The slurry sample had a yield reduction of about 13% under similar operating conditions, except that it was fixed on silica gel granules^59^. Exposure to visible light showed a significant effect of cobalt promoter on increasing the performance of TiO_2_ in the decomposition of amoxicillin^60^, which enhanced the efficiency by as much as 70% increase compared to TiO_2._ In another work, bismuth (as a promoter) and platinum were used to amplify TiO_2_ in the photo-catalytic analysis of amoxicillin (10 ppm), which achieved an efficiency of 87% in 120 min under visible light^61^.

**Table 3:** Comparison of this work with the recent reports on amoxicillin degradation.

Photo-catalyst	S (Slurry)	Amoxicillin Concentration	Time (min)	Light Intensity	Yield	Ref.
	I (Immobilized)					
TiO_2_ (P25)	I	50 ppm	500	300 W	80%	^56^
TiO_2_/WO_3_	I	100 ppm	–	550 kJ/ m^2^	64%	^57^
Anatase TiO_2_	S	104 mg/L	300	6 W	70.9%	^58^
Anatase TiO_2_	I	100 ppm	300	10 W	57.7%	^59^
TiO_2_ (P25)	S	10 ppm	60 (min in dark) 300	670 W/m^2^visible	16%	^60^
TiO_2_					21%	
Co/TiO_2_					94%	
1% Pt/5% Bi/TiO_2_	S	10 ppm	120	300 WHalogen tungsten	87.67%	^61^
TiO_2_/ZnO/GOx Bio-photo-catalyst	I	50 ppm	60	0.25 W/cm^3^	88%	Present Work “Optical study” Section

In the present work, a TiO_2-_ based bio-photo-catalyst coated capillary has been attained by modification of TiO_2_ with glucose oxidase and oxide nanoparticles, and the complete decomposition of amoxicillin was irradiated with a 0.25 W/cm^3^ UV-light source for 120 min at 50 ppm.

ZnO is dopant and it is not as influential as TiO_2_ is. Therefore, its effect on the result is not that prominent. However, it is worth studying. Two findings are concluded from Table 4. First, it recommends that the microfluidic system is a better environment for ZnO synthesis. Second, size distribution is the key property for achieving higher efficiency. In another word, by tuning the flow rates, a desired size distribution and uniformity can be attained. For instance, once the volumetric flow rate of NaOH is set at 50 µl/min and ZnCl_2_ at 25 µl/min, a greater amoxicillin degradation and narrower size distribution are attained than the case in which both reagents collide with each other at 50 µl/min flowrate.

**Table 4:** Comparative systems for ZnO synthesis based on performance evaluation.

Operational Condition of photo-catalytic test	Amoxicillin Degradation efficiency over TiO_2_/ZnO/GOx		
	Bulk synthesis of ZnO at 100 °C	Room Temperature synthesis of ZnO in Spiral Microfluidic device	
		Q _NaOH_ = Q _ZnCl2_ = 50 µl/min	Q _NaOH_ = 50 µl/min Q _ZnCl2_ = 25 µl/min
A: pH = 5.5, B: UV light intensity = 0.145 W/m^3^, C: Amoxi. Concentration = 65 ppm, D: Time = 75 min, E: Ti foil area = 1.5 cm^2^, F: ZnO/GOx = 0.125, G: Glucose/GOx = 0.2	85.2%	98.8%	92.8%

## Conclusion

In this work, the synthesis time in the microfluidic system is cut from hours to minutes (for TiO_2_ each cycle is 25 min and for ZnO nanoparticles, it is 15 min). XRD results indicate that at a low temperature of 60 °C anatase formation is successfully attained. FTIR analysis indicate that ZnO nanoparticles which are synthesized in bulk and microfluidic systems are identical with respect to chemical bonds (ZnO stretching vibrations, Zn–OH peaks, C–OH bond, C=O groups, and O–H stretches). The biological activity of GOx enzyme is confirmed by Raman spectroscopy and FTIR tests that show the H_2_O_2_ generation. The best temperature to synthesize TiO_2_ is 70 °C; according to SEM results the nanoparticles are the finest and the size distribution is narrowest. Furthermore, the highest apparent rate constant of 0.035 min^-1^ and the highest yields of amoxicillin degradation (100% for 10 ppm, 93.9% for 30 ppm, and 87.8% for 50 ppm) ascribed to this sample. The findings of this work pave the way for upcoming researchers deciding to work on the development of new bio-photo-catalysts and their applications.

## Data Availability

All data generated or analyzed during this study are included in this published article.
